# Functional properties of an ensemble of candidate germline-encoded precursors of the anti-MPER antibody 4E10

**DOI:** 10.1186/1742-4690-9-S2-P344

**Published:** 2012-09-13

**Authors:** K Finton, B Larmen, K Larimore, A Stuart, D Friend, T Vanden Bos, P Greenberg, S Elledge, L Stamatatos, R Strong

**Affiliations:** 1 Fred Hutchinson Cancer Research Center, Seattle, WA, USA; 2 Harvard Medical School, Boston, MA, USA; 3 University of Washington, Seattle, WA, USA; 4 Seattle Biomedical Research Institute, Seattle, WA, USA

## Background

We have previously engineered computationally-designed ‘epitope-scaffold’ constructs for the broadly neutralizing, MPER-specific antibody 4E10, consisting of the epitope grafted as a structural unit onto non-HIV scaffold proteins for optimal presentation during immunization. 4E10 epitope-scaffolds display dissociation constants for mature 4E10 ranging down to picomolar values and can elicit epitope-specific responses during immunization. Sera from immunized animals failed to potently neutralize HIV, at least partially due to differences between human and non-human germline repertoires. Successful use of epitope-scaffolds as vaccine immunogens will require optimizing interactions with both the mature antibody target and appropriate precursors, while preserving or generating neutralization potency during maturation.

## Methods

Since a unique germline-encoded precursor sequence for 4E10 cannot be unambiguously assigned, we have generated an ensemble of the twelve likeliest candidates in order to study their functional and recognition properties. Interaction parameters between mature and candidate germline-encoded precursor (CGP) antibodies for engineered epitope-scaffolds, peptides, membrane components and HIV proteins were determined in surface plasmon resonance analyses and three-dimensional structures of free and ligand-complexed forms were determined by x-ray crystallography. Neutralization potencies were determined and polyspecificity and autoreactivity were analyzed with large-scale peptide arrays.

## Results

Unlike other anti-HIV CGPs, which display negligible affinities for HIV-related ligands, 4E10 CGPs display affinities for epitope-scaffolds which, while 1,000- to 100,000-fold weaker, still reach into the nanomolar range. Structural studies show remarkable conservation of recognition mechanisms while functional studies show retention of anti-HIV activity. Polyspecificity of both mature and CGP forms is very limited, but the significant autoreactivity of mature 4E10 appears directed to specific targets.

## Conclusion

The functional gap between mature 4E10 and its CGPs is narrower than in other HIV-related systems. Strategies based on our results can be proposed to generate mature- and CGP-specific epitope-scaffolds for use in prime-boost vaccinations to target specific CGPs with desirable functional properties while potentially avoiding autoreactivity.

